# Interaction between honeybee mandibles and propolis

**DOI:** 10.3762/bjnano.13.84

**Published:** 2022-09-14

**Authors:** Leonie Saccardi, Franz Brümmer, Jonas Schiebl, Oliver Schwarz, Alexander Kovalev, Stanislav Gorb

**Affiliations:** 1 University of Stuttgart, IBBS, Research Unit Biodiversity and Scientific Diving, Stuttgart, Germany; 2 Department Biomechatronic Systems, Fraunhofer Institute for Manufacturing Engineering and Automation IPA, Stuttgart, Germany; 3 Department Functional Morphology and Biomechanics, Zoological Institute, Kiel University, Kiel, Germany

**Keywords:** adhesion, *Apis mellifera*, bee mandibles, honeybee, propolis

## Abstract

In a biomimetic top-down process, challenging the problem of resin deposition on woodworking machine tools, an adequate biological model was sought, which hypothetically could have developed evolutionary anti-adhesive strategies. The honeybee (*Apis mellifera*) was identified as an analogue model since it collects and processes propolis, which largely consists of collected tree resin. Propolis is a sticky substance used by bees to seal their hive and protect the colony against pathogens. In spite of its stickiness, honeybees are able to handle and manipulate propolis with their mandibles. We wanted to know if beneficial anti-adhesive properties of bee mandibles reduce propolis adhesion. The anatomy of bee mandibles was studied in a (cryo-)scanning electron microscope. Adhesion experiments were performed with propolis on bee mandibles to find out if bee mandibles have anti-adhesive properties that enable bees to handle the sticky material. A scale-like pattern was found on the inside of the mandible. Fresh mandibles were covered with a seemingly fluid substance that was at least partially removed during the washing process. Propolis adhesion on bee mandibles was measured to be 1 J/m^2^ and was indeed significantly lower compared to five technical materials. Propolis adhesion was higher on mandibles that were washed compared to fresh, unwashed mandibles. Results indicate that the medial surface of the mandible is covered with a fluid substance that reduces propolis adhesion. First results suggested that the surface pattern does do not have a direct effect on propolis adhesion.

## Introduction

Nature has solutions for many of the problems and challenges humans face, since different creatures have adapted to similar challenges during their evolution. A common problem category in technology is unwanted adhesion; whether it is cake sticking to the cake tray, smeared glasses, or industrial production processes where unwanted build-up occurs. Specifically, this problem also occurs in woodworking processes, where escaping resins contaminate the cutting edge of the tools and, subsequently, blunt the carbide cutting edges through abrasion. This was the starting point of a biomimetic top-down development process that led to the anatomical investigation of honeybee mandibles.

The hypothesis is that animal species that regularly have close contact with resinous plants or even actively harvest resins may have developed counter-stickiness strategies. This is because animals that permanently stick to a plant are deprived of further reproduction, which exerts a significant evolutionary selection pressure. The honeybee (*Apis mellifera*) was chosen as a possible model because it collects and processes propolis, a sticky and ductile material, that is created by mixing plant resins, wax, and other substances. Similar to tree resins, which contaminate the surfaces of processing tools, propolis also adheres well to different types of surfaces [1].

Bees use propolis as a building material, for example to seal cracks and smooth out the internal walls of the hive [2–3]. In addition to its mechanical functions, propolis also has chemical properties that protect the colony and contribute to the social immunity [4]. Because of its strongly adhesive nature, propolis is sometimes referred to as bee glue [2,5].

### Anatomy of bee body parts handling propolis

The mouthparts of bees are used to collect food, water, and resin [6]. They are therefore adapted to extract liquids as well as to grasp and manipulate objects [5–6]. The mouthparts consist of labrum, mandibles, maxillae, and labium. The labium is used as a tongue to take up liquids while the mandibles are responsible for tasks involving grasping [5–6]. The mandibles of the bee are instrumental in processing propolis, but they are also used for other tasks such as biting through cell caps and modelling wax [7]. The mandibles, sometimes also called jaws, are situated laterally on the lower part of the bee’s head (see Figure 3) and operate transversely [7–8]. Mandibles of worker bees are spoon-shaped and differ from those of the queen and drones, which have a more pointed apex and a subapical notch [7–8]. The medial surface of the mandible has not been studied in detail before but has been described as concave and ridged [8]. The cuticle of mandibles is particularly strong due to bonding of long chitin chains and sclerotization (crosslinking of proteins in the cuticle) [6].

### Foraging and handling of propolis

Worker bees exhibit a division of labour based on age (polyethism) [9] in which duties such as brood rearing are usually performed by younger bees while middle-aged bees process propolis in the hive and older bees collect resins [10]. Honeybees mainly forage resins from buds, though they also have been reported to collect resin from tree barks and fruit surfaces [4,11–12]. It is also unclear how exactly propolis bees detect suitable resin sources. Some plants might release chemicals that are picked up by bees [4,13]. Some bees have also been observed probing different plants before collecting resin [14]. When they have found a resin source, they start by first biting off a piece of resin and working it with the mandibles [4,15]. The front legs are used to pass the resin from the mandibles to the mid legs and then from there to the corbiculae [15–16]. Once both corbiculae are fully loaded with resin, the propolis bee returns to the hive, where other so-called cementing bees unload it using their mandibles and subsequently mix it with wax [4,15]. The resulting material is now called propolis [4]. Cementing bees then attach propolis to the walls of the hive and smooth it out with their mandibles [10–11]. The mandibles play the most important role in the oral apparatus of bees when processing resin and propolis.

### Anti-adhesive strategies in biology

One might expect that highly adhesive substances such as resin and propolis stick to the bee’s mouthparts and legs rendering them unusable. But bees are able to process these sticky substances and apparently without being negatively affected by them. It is therefore possible that anti-adhesive properties minimize adhesion of resins and propolis to their body parts. Various anti-adhesive strategies have been found in nature. Different mechanisms can lead to low adhesion. Possible strategies to reduce adhesion on the insect cuticle, as suggested in [16], are specific surface chemistry, surface microstructures, an easy-to-break solid layer preventing strong bonding, or a fluid layer providing cohesion failure.

### Resin adhesion on stingless bees

One example for possibly anti-adhesive surfaces that is especially relevant to this work, are Bornean stingless bees (Hymenoptera, Meliponini). Similar to honeybees, stingless bees harvest resin to build their nests and defend it against intruders [17]. Resin can easily be removed from the mandible surface without leaving residue behind [18–19]. It was therefore suggested that stingless bees might temporarily lubricate their mandibles to reduce resin adhesion [19]. In [20], it was observed that stingless bees were not able to handle propolis until they reached a certain age. They suggested that this might be due to gland development and production of glandular substances [20].

### Objective

The main objective of this work was to understand the interaction between honeybee mandibles and propolis. The hypothesis is that beneficial anti-adhesive properties have evolved to handle propolis without being hindered by resin contamination. Possibly, these anti-adhesive properties could serve as an inspiration to help solve problems such as resin contaminating woodworking tools.

The anatomy of bee mandibles has previously been described [6–8,19], though not with a detailed focus on the medial surface of the mandible tip (see below in Figure 3B). However, this part is especially interesting in terms of propolis processing, since this surface is in intensive contact with the sticky material during biting and shaping it. Therefore, the anatomy of bee mandibles was studied, focusing on the medial surface of the mandible tip to find possible reasons for reduced adhesion. Once physical and adhesive propolis properties as well as the anatomy of bee mandibles were characterized, it was possible to test whether propolis adhesion is indeed reduced on bee mandibles. Therefore, adhesion experiments with propolis were performed on bee mandibles.

## Materials and Methods

### Propolis and insects

#### Propolis

**Figure 1:**
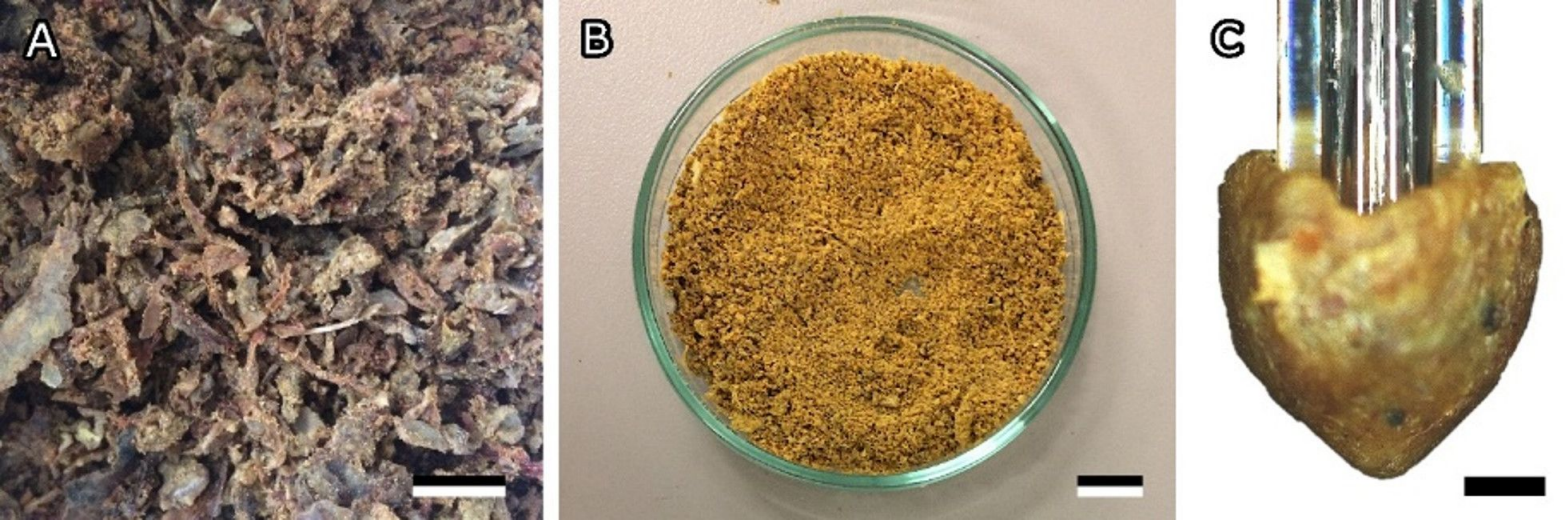
Bee propolis. (A) Raw propolis as collected from the hive. (B) Homogenised propolis powder. (C) Cone-shaped propolis sample used for adhesion tests. Scale bar: 1 cm (A, B), 500 µm (C). Figure 1 was reproduced from [1] (© 2021 Saccardi, Schiebl, Weber, Schwarz, Gorb and Kovalev, published by Frontiers, distributed under the terms of the Creative Commons Attribution 4.0 International License, https://creativecommons.org/licenses/by/4.0).

Raw propolis provided by private beekeeper Dr. Oliver Schwarz (Stuttgart, Germany) (Figure 1A) was harvested and homogenised as described in [1]. For homogenisation, propolis chunks were mixed, frozen finely ground and subsequently stored at −20 °C (Figure 1B). The pulverizing procedure was based on a method that is used to produce propolis extract [21]. To prevent contamination, propolis was only handled wearing gloves cleaned with ethanol (Rotipuran®, ≥99.8%, p.a., Carl Roth GmbH & Co. KG, Karlsruhe, Germany).

#### Insects

Adult worker bees (*Apis mellifera*) were collected in gardens in Kiel (Germany) in July 2019 and immediately used for experiments. They are hereinafter referred to as „nectar collectors“. In October 2018, bees were taken directly from the hive (Stuttgart, Germany). They are further named „hive bees“. They were kept in a cage and provided with water and honey until they were prepared for experiments. The remaining bees were frozen and stored at −20 °C for three months to be used in further experiments.

Honeybees (*Apis mellifera*), returning to the hive with resin attached to their pollen baskets, were caught and subsequently frozen in September and October 2018. These resin-collecting bees will be henceforth referred to as “propolis bees”.

### Imaging and structural studies

Bee mandibles were prepared and subsequently examined with binoculars, a scanning electron microscope (SEM), and a confocal 3D laser scanning microscope in order to identify anatomy and surface structure.

#### Anatomy of the honeybee mandible

**Figure 2:**
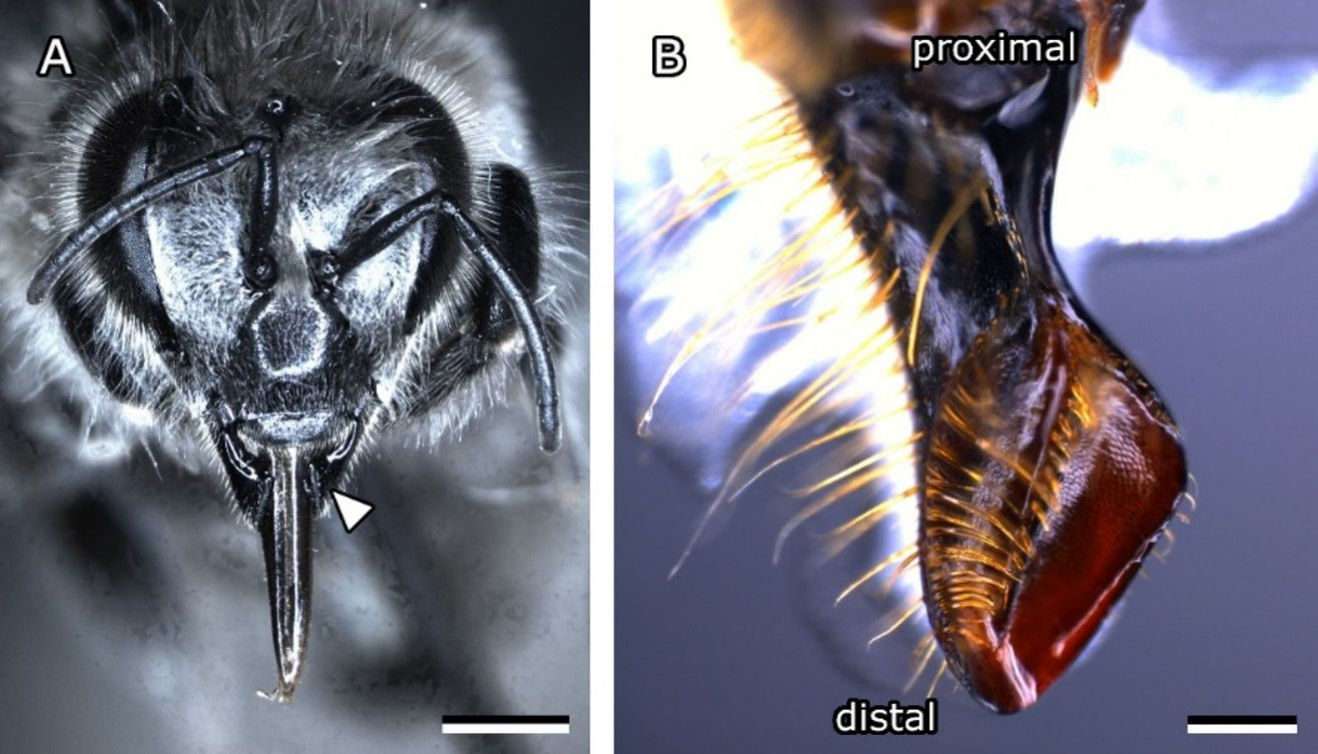
Head and mandible of a worker bee. (A) Frontal view of a honeybee head with an arrow pointing to the outside of the left mandible. Scale bar: 1 mm. (B) Medial surface of the left mandible of a worker bee. Proximal and distal ends are marked. Scale bar: 200 µm.

Mandibles of all collected bees were prepared under binoculars by carefully separating them from the insect’s head with a scalpel (Figure 2). General morphology, structures, and contamination of every prepared mandible were studied with a binocular microscope (Leica M205 A, Leica Microsystems GmbH, Wetzlar, Germany) equipped with a camera (Leica DFC420) prior to further experiments. For the following anatomical studies, mandibles of nectar collector and hive bees were then washed with acetone and water. They were air-dried, mounted on holders (inside up), sputter-coated with a 10 nm thick layer of gold–palladium and studied using a SEM (Hitachi S-4800, Hitachi High-Technologies Corp., Tokyo, Japan) at 3 kV accelerating voltage. Images of the spoon-shaped mandible tip were taken systematically and later assembled into one high resolution image. Higher magnified pictures were taken in characteristic areas of the mandible surface. For one additional experiment, instead of air-drying, four washed mandibles were dried using a critical-point-drier (Leica EM CPD300) and subsequently studied in the SEM.

#### Surface structures on bee mandibles

Surface structures on mandibles were studied in the SEM as described above. Additionally, the medial surface profile of the mandible was studied on fresh, chloroform-washed specimens fixed to a glass slide using a drop of a melted mixture of 50% paraffin wax and 50% colophony. The mandibles were examined with a confocal 3D laser scanning microscope (Keyence VK-X250; Keyence Corporation, Osaka, Japan). The MultiFileAnalyser software (Version 1.2.6.106, Keyence Corporation, Osaka, Japan) was used to measure profiles and the structural dimensions in different areas of the mandible.

#### Surface structures on propolis bee mandibles

Freshly defrosted and prepared mandibles of propolis bees were studied in the cryo-SEM in the frozen state so the resin contaminations did not dry out. They were placed on holders (medial surface up), rapidly frozen on the table in the preparation chamber at −140 °C, sputter coated with gold palladium (3 nm), and studied at −120 °C with a cryoSEM (Hitachi S-4800, Hitachi High-Technologies Corp., Tokyo, Japan) equipped with a cryopreparation system (Gatan ALTO 2500, Gatan, Inc., Abingdon, UK) at 3 kV accelerating voltage.

#### Image processing

SEM images were processed using Gimp, version 2.10.14. All adjustments were applied to the whole image. Color levels, contrast, and brightness were adjusted, and scale bars and other labels were added. Profiles measured with the confocal 3D laser scanning microscope were digitized using WebPlotDigitizer (version 4.2, https://automeris.io/WebPlotDigitizer, Ankit Rohatgi, San Francisco, USA).

### Investigation of propolis adhesion on mandibles

#### Insect preparation for adhesion tests

After insects for experiments were caught, they were placed and stored in the freezer at −20 °C for a minimum of 15 min and up to many months. The mandibles were prepared as described above (Figure 2). Without further treatment a mandible was then glued, inside facing up, to a glass slide using a drop of a melted mixture of 50% paraffin wax and 50% colophony to avoid unwanted movement during adhesion experiments. The spoon-shaped, distal part of the mandible was oriented using a binocular microscope so that the flat area near the sharp edge of the mandible was parallel to the glass surface. Adhesion experiments on mandibles were carried out immediately after fixing the mandible to the slide to avoid material desiccation.

#### Test method

**Figure 3:**
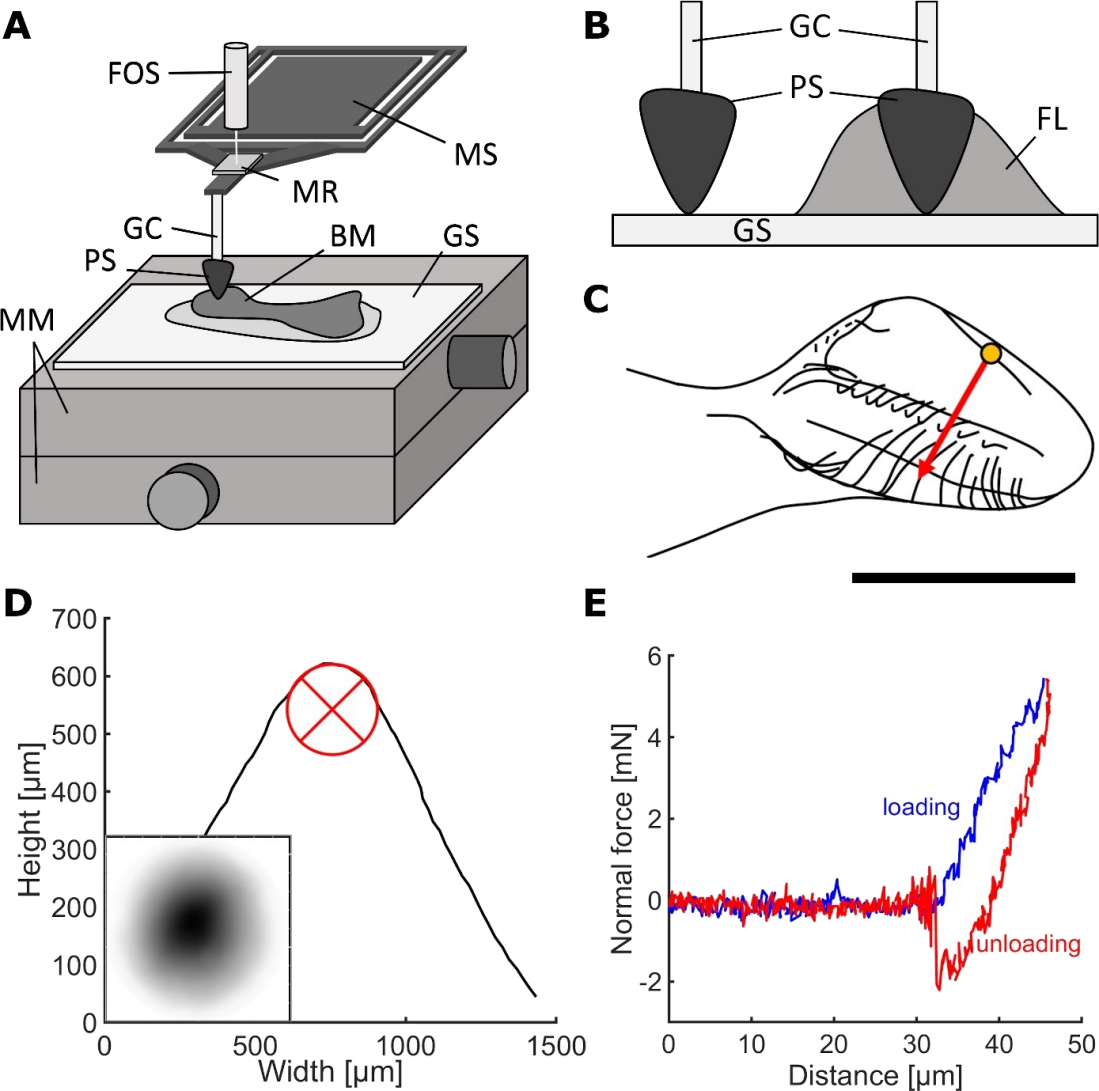
Adhesion experiments. (A) Experimental set-up for adhesion testing with the Basalt-01 mechanical tester (Tetra GmbH). (B) Propolis contact in the presence of fluid (mineral oil) and without fluid. (C) Schematic of a bee mandible. Adhesion measurements were conducted along the arrow, the circle represents the size of the contact area. Scale bar: 500 µm. (D) 3D profile of a propolis sample. The inscribed circle was used to estimate the tip radius. The small subimage depicts the sample topography with darker areas being higher than lighter areas. (E) Typical force–distance curve obtained from adhesion experiments. BM, bee mandible (optional); FL, fluid; FOS, fibre-optic sensor; GC, glass capillary; GS, glass slide or other substrate material; MM, micro-manipulators; MR, mirror; MS, metal spring; PS, propolis sample. Figure 3A was adapted and Figure 3B, D, and E were reproduced from [1] (© 2021 Saccardi, Schiebl, Weber, Schwarz, Gorb and Kovalev, published by Frontiers, distributed under the terms of the Creative Commons Attribution 4.0 International License, https://creativecommons.org/licenses/by/4.0).

Adhesion experiments were performed in a manner similar to that in [1]. Just before each adhesion experiment, a small amount of homogenised propolis powder was defrosted and kneaded into a homogeneous mass. Cone-shaped propolis samples with a spherical tip were subsequently formed by hand wearing ethanol-cleaned gloves (Figure 1C). The topography of the sample was analysed using a 3D optical profilometer (Keyence VR 3100; Keyence Corporation, Osaka, Japan). The profile of the sample was measured along five lines arranged in a star shape going through the highest point of the tip. To estimate the radius at the sample tip, a circle was fitted to the sample profiles in the five orientations (Figure 3D). The radii of the circles were measured and then averaged.

The effective elastic modulus and pull-off force of propolis were measured with a microforce measurement device (Basalt-01; Tetra GmbH, Ilmenau, Germany) [22–24]. The device mainly consists of micromanipulators as a platform holding the substrate material, a metal spring (springs with spring constants of 618 N*/*m and 539 N*/*m were used) and a fibre-optic sensor (Figure 3A). The piezo drive moves the spring down to load and up to unload the sample. A shortened glass capillary (5 µL micropipet Blaubrand^®^ IntraEND, Brand GmbH & Co. KG, Wertheim, Germany) was attached to the metal spring with cyanoacrylate glue (5925 Ergo^®^ Elastomer–Kisling GmbH, Bad Mergentheim, Germany). The freshly formed, cone-shaped sample of propolis was then mounted on the tip of the capillary without any additional glue. The propolis sample was brought into contact with the substrate and retracted from the surface as soon as the load force reached 5 mN. The load was chosen to resemble the force applied by bees when handling propolis. As no studies exist on mandibular forces and pressures of honeybees, pressures measured at the tip of mandibles of predacious coleoptera [25] where used as a reference point. Tip pressures were calculated as suggested by [25]:


[1] $$P=\frac{F_{\text{a}}}{A},$$

where *F*_a_ is the applied force and *A* is the contact area obtained from the contact radius.

With each propolis sample, a set of ten single measurements was performed, each on a different spot on the spoon-shaped tip of the bee mandible, starting at the sharp edge and moving towards the hairy edge (Figure 3C). Another measurement with an extended contact time of 60 s was performed on the flat area of the bee mandible. Afterwards, six reference measurements were carried out on a smooth, clean glass surface (standard microscopy slides (soda lime glass); Carl Roth GmbH & Co. KG, Karlsruhe, Germany) with the same propolis sample.

Experiments were carried out at room temperature (24.00 ± 0.53 °C) and a relative humidity of 36.80% ± 9.0%. For every condition, 50–150 single tests were performed on different spots of the mandible (*N* = 5–15 propolis samples and mandibles, *n* = 10 measurements on mandible per sample). After the adhesion experiments, the substrate material was examined under a binocular microscope (Leica M205 A) in order to find possible propolis residues/prints in the contact area. Adhesion tests on fresh mandibles were performed in July and November. As a reference, propolis adhesion on bee mandibles was compared to propolis adhesion measured on glass under dry and fluid conditions [1]. For the reference experiments in fluid conditions, a drop of oil (mineral oil, light, Sigma-Aldrich, St. Louis, USA) was placed on the glass surface as described in [1] and shown in Figure 3B. Oil was chosen because we assumed that the mandibles surface might be oily.

### Evaluation of a potential coating of the mandibles

As previously suggested, a fluid layer covering the mandible surface could be a strategy to reduce adhesion [16]. Therefore, fresh, untreated bee mandibles were studied in the frozen state, as cryo-SEM has been reported to be a successful method for visualizing biological fluids such as lipids and water-based solutions [26]. Cryo-SEM was performed as described in section “Surface structures on propolis bee mandibles”.

Additionally, visualization of the mandible cuticle and any additional surface layers was performed using cryo-SEM on fresh fractures of untreated bee mandibles. Mandibles were tightly clamped into a metal holder and frozen in the preparation chamber (−140 °C). The samples were then fractured within the preparation chamber by cutting off a part of the mandible tip using a cold scalpel blade mounted on a user-controlled handle. Fractured samples were then sputter coated and examined in a frozen state as described above.

Where possible, the contact angle of the substance coating the mandible surface was measured on SEM images of fresh fractures of untreated bee mandibles using Gimp, version 2.10.14. For this purpose, two measurement lines were drawn: one along the surface of the cuticle and one along the surface of the substance on top of the cuticle. The angle was then measured between the two lines.

Furthermore, different solvents were used to wash fresh mandibles in order to try removing any potential surface coating. Mandibles were washed in an ultrasonic bath with distilled water for 10 min or with either chloroform (Rotisolv^®^ HPLC stabilised with 1% ethanol, Carl Roth GmbH & Co. KG), or acetone (Rotipuran^®^ ≥99.8%, p. a. ACS ISO, Carl Roth GmbH & Co. KG) for 5 min followed by 5 min washing with distilled water. Distilled water was used as an agent for removing hydrophilic substances from the surface of bee mandibles. Chloroform and acetone are known to remove hydrophobic substances and the wax layer from mandibles [27]. The ultrasonic bath facilitates the dissolving procedure and particle removal from the surface.

To visualise the effect of washing, dry untreated mandibles and mandibles treated with the different solvents were examined using a SEM as described before (section “Anatomy of the honeybee mandible”). Mandibles washed with chloroform and water were studied in the cryo-SEM as described above for untreated mandibles.

#### Adhesion on washed bee mandibles

To test the effect of potential surface coatings on propolis, adhesion experiments with propolis were performed on mandibles washed with the different methods as described for untreated bee mandibles (section “Investigation of propolis adhesion on mandibles”).

### Adhesion on resin replicas of bee mandibles

In order to test how shape and surface structures of the mandibles affect adhesion, replicas of bee mandibles were made using a two-step moulding method [26,28]. The method allows one to replicate the surface structure with nanometre precision. Replication substitutes chemical complex and heterogeneous biological surfaces with a well-studied epoxy surface. In this way the effect of surface chemistry on adhesion was excluded. First, washed mandibles fixed to glass slides, as described above (section “Investigation of propolis adhesion on mandibles”), were covered with a two-component dental wax (Affinis light body, ISO 4823, polyvinylsiloxane, Coltène Whaledent AG, Altstätten, Switzerland). After the wax was cured, the mandibles were removed from the mould, the cavity was filled with Spurr epoxy resin (Spurr’s low viscosity kit; Plano, Wetzlar, Germany, composition: vinyl cyclohexene dioxide 10 g, diglycidyl ether of polypropylene glycol 6 g, nonenyl succinic anhydride 26 g, and S-1 dimethylaminoethanol 0.4 g [29]) and covered with a smooth sheet of dental wax to create a level surface for the mandible replica to stand on. The resin mandibles were cured at 70 °C for 48 h, demoulded and fixed to a glass slide using double-sided adhesive tape. A binocular microscope (Leica M205 A) was used to visually check whether the features of the real mandibles, especially the surface structures, were successfully replicated in the artificial mandibles.

Adhesion experiments with propolis were carried out as described for real bee mandibles (section “Investigation of propolis adhesion on mandibles”). For mandible replicas, reference measurements were performed on the smooth resin substrate. 50–60 single tests were performed on different spots of the mandible replica (*N* = 5–6 propolis samples and mandibles, *n* = 10 measurements on mandible per sample).

### Data analysis and statistics

Adhesion experiments were evaluated as described in [1] using Matlab, version R2015b. The unloading part of force–distance curves (Figure 3E) acquired from adhesion experiments was fitted according to the JKR theory [30]:


[2] $$a^{3}=\frac{3R}{4E}\left[F_{\text{a}}+3\pi R\Delta \gamma +\sqrt{6\pi RF_{\text{a}}\Delta \gamma +{\left(3\pi R\Delta \gamma \right)}^{2}}\right],$$

where *a* is the contact radius, *F*_a_ is the applied load, *R* is the tip radius, and *E* and ∆γ are the effective elastic modulus and the work of adhesion, respectively. The work of adhesion ∆γ is the energy per unit of area needed to separate two bodies in contact. It was chosen as a measure of adhesion because it is independent of the contact area. To characterize viscoelastic properties of propolis, a generalized Maxwell model was used [31]. The viscosity of the sample was estimated from experimental force curves using the following equation [32–33]:


[3] $$F_{\text{a}}=\frac{4\sqrt{R}d^{1.5}}{3\left(1-\nu^{2}\right)}\left(E_{\infty }+E_{1}e^{-\frac{E_{1}t}{\eta_{1}}}+E_{2}e^{-\frac{E_{2}t}{\eta_{2}}}\right),$$

where *d* is the displacement, *t* is the time under load, *E*_∞_/*E*_1_/*E*_2_ and η_1_/η_2_ are the Young’s moduli and viscosities of the static and the two dynamic components, and ν is the Poisson ratio assumed to be equal to 0.49 [33].

The data were statistically analysed using the software R, version 3.6.1. Data was tested for normal distribution and variance homogeneity using Kolmogorov–Smirnov and Levene's tests, respectively. The comparison of propolis adhesion under different conditions and on different substrates was performed with a one-way ANOVA and a pairwise multiple comparison procedure (Tukey test). An unpaired two-sample *t*-test was performed to compare the mean Young’s modulus of propolis at 24 and 26 °C. Correlation analysis of Young’s modulus and work of adhesion obtained from adhesion experiments was performed by calculating the Pearson correlation coefficient.

## Results

### Honeybee mandible

When bees handle propolis, the mandibles are the first surfaces that will come into contact with plant resin or propolis. They were thus analysed using various imaging methods.

#### Anatomy of the bee mandible

**Figure 4:**
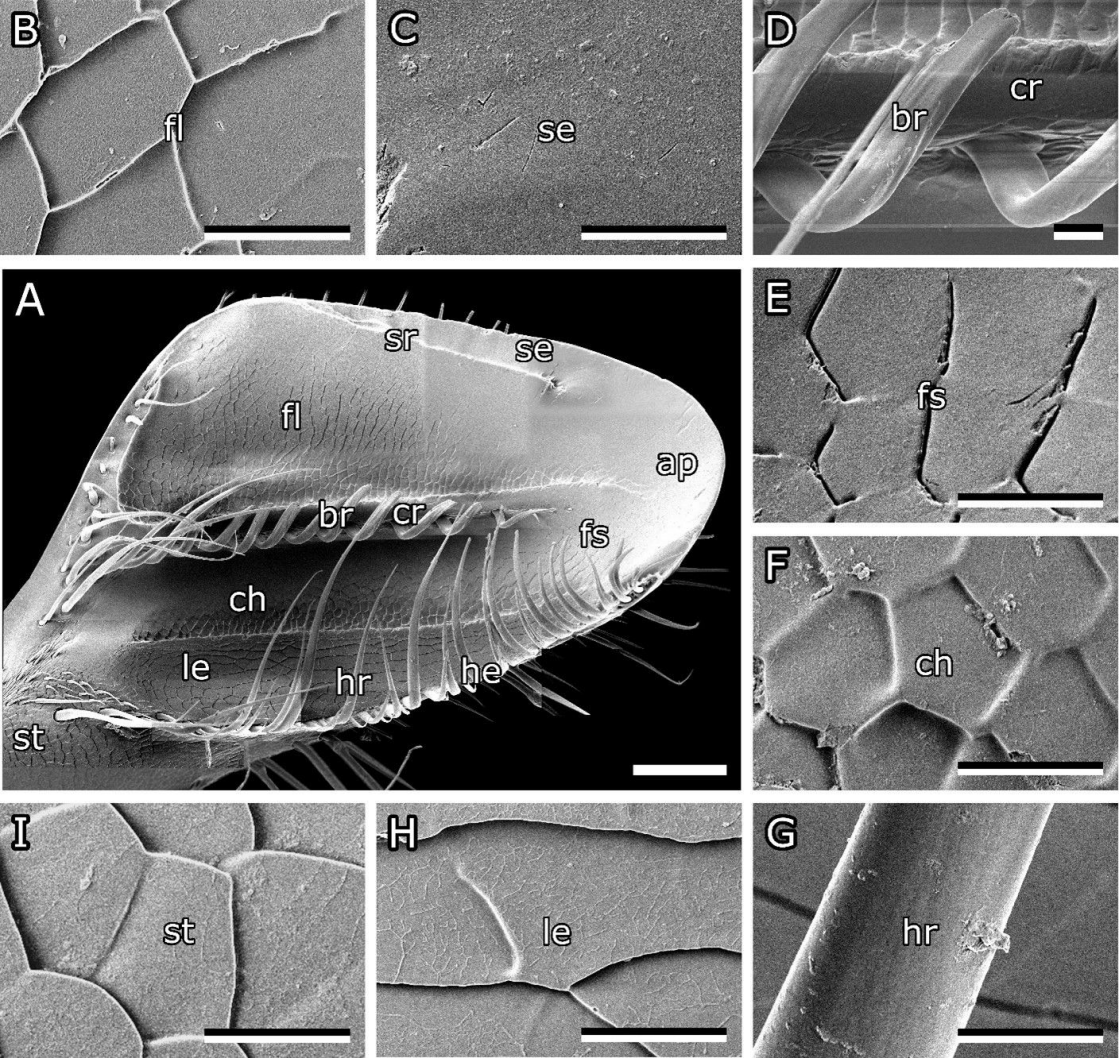
Anatomy of the bee mandible. (A) Medial surface of left bee mandible. Characteristic features are labelled. (B–I) Higher magnified images of areas labelled in (A). (B) Scales in flat area. (C) Smooth surface near the sharp edge. (D) Curved bristles along the central ridge. (E) Fading structures towards the apex. (F) Scales in channel area. (G) Hair growing on the hairy edge. (H) Oblong scales on the ledge. (I) Scales on the stem of the mandible. ap, apex; br, bristles; ch, channel area; cr, central rigde; fl, flat area; fs, faded structures; hr, hair; he, hairy edge; le, ledge; se, sharp edge; sr, small ridge; st, stem. Scale bars 100 µm (A), 10 µm (B–I).

The anatomy of the worker bee mandibles (*Apis mellifera*) was studied using a binocular microscope and SEM on acetone-cleaned specimens. Mandibles have a strong stem at the proximal end and a concave, spoon-shaped tip at the distal end (Figure 2B). The medial surface of this tip was examined more extensively because it is used by bees to process and form propolis (Figure 4A). The spoon-shaped tip tapers towards the distal apex. On one side it exhibits an edge densely covered by smooth, long, and flexible hairs (hairy edge), reaching over half of the mandible medial surface (Figure 4A,G), while the edge on the opposite site is sharp and hairless (sharp edge). Though, short, thin hairs grow on the mandible outside close to the sharp edge. A ridge runs along the centre of the medial surface of the mandible tip (central ridge) fading towards the apex. It is spiked with stiff, slightly grooved bristles that are curved towards the sharp edge (Figure 4A,D).

An elevated ledge lies next to the hairy edge. The area between the central ridge and the ledge seems to form a channel that runs from the stem to the apex. A groove runs along the inside of the stem merging into the channel area on the tip (Figure 3A,F). On the other side of the central ridge (Figure 3A,D), there is a flat area reaching up to the sharp edge. A smaller ridge starts at the proximal end of the sharp edge and is angled towards the apex but stopping at about the same height as the bristles and hairs. The outside of the mandible is covered with hairs evenly.

#### Surface structures on bee mandibles

SEM micrographs also revealed that bee mandibles are covered with anisotropic scale-like micropatterns (Figure 4). Most of the scales on the medial surface of the mandible were pentagonal or hexagonal, though the shape and proportions varied depending on the area they cover. The scales in the flat area measured 18.1 ± 2.4 µm/9.55 ± 1.3 µm at their minimum/maximum width, respectively (Figure 4B). However, closer to the apex and the sharp edge, the structures were less pronounced and eventually seemed to fade completely (Figure 4C,E). In the channel area, the structures were rounder (9.34 ± 0.77 µm/8.21 ± 0.82 µm) and had blunt edges (Figure 4F). Scales on the ledge on the other hand had an oblong shape (31.67 ± 9.34 µm/9.05 ± 2.25 µm) and were oriented parallel to the hairy edge (Figure 4H). The scales on the outside of the mandible are more rounded and form rows of scales that are not clearly separated (12.62 ± 2.71 µm/12.72 ± 1.49 µm) (Figure 4I).

#### Scale profile

**Figure 5:**
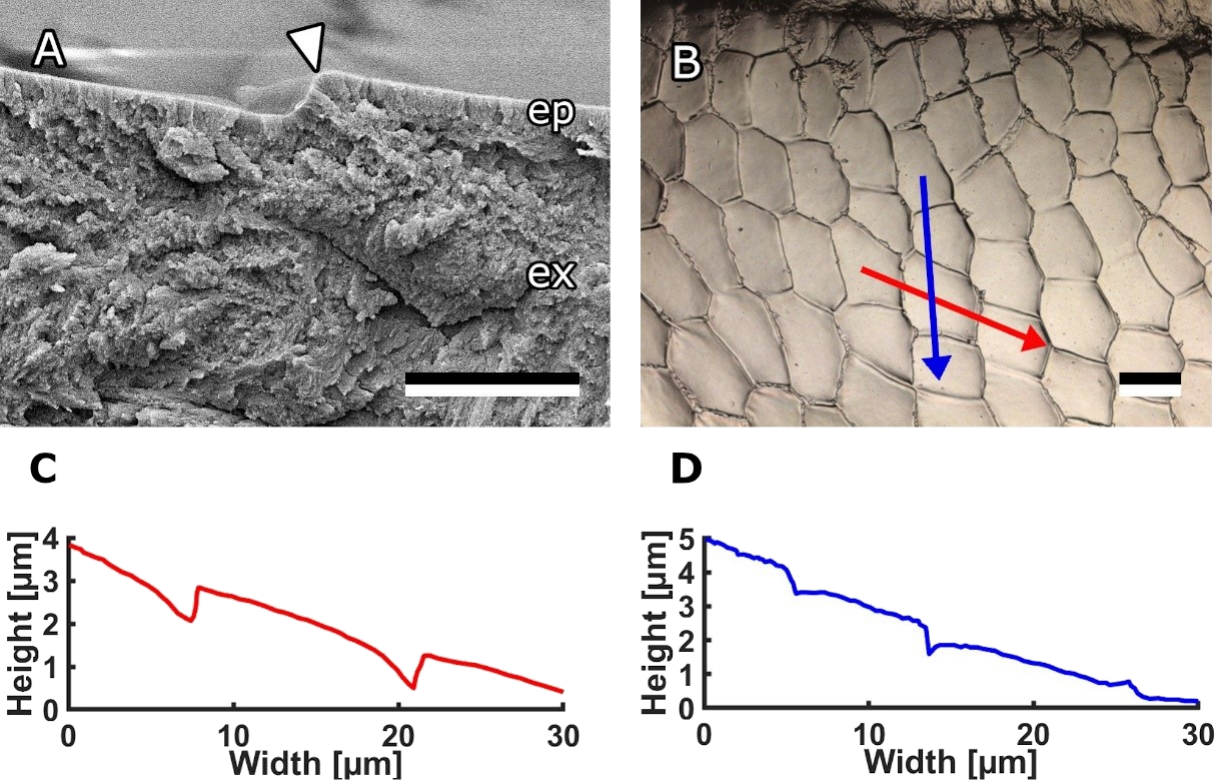
Profile of scales on bee mandibles. (A) Cryo-SEM micrograph of mandible cuticle. The arrowhead indicates the step between two scales. (B) 3D laser scanning microscope image of medial surface of the mandible in the channel area. (C) Profile of scales in along the red arrow in (B). (D) Profile of scales along the blue arrow in (B). Scale bars: 2 µm (A), 10 µm (B).

A 3D laser scanning microscope was used to study the topography of these structures on the medial surface of the mandible (Figure 5). The scales are flat or slightly convex and overlap or form steps between them. The step height between the scales was measured to be 0.71 ± 0.26 µm in most areas of the mandible. Though, in areas, were the scale pattern seemed to fade, the step heights were smaller (0.05–0.30 µm). In the flat area the scales descended towards the apex, while scales in the channel area were not oriented in a clear direction and scales on top of the ledge were descending towards the hairy edge.

#### Propolis bees

**Figure 6:**
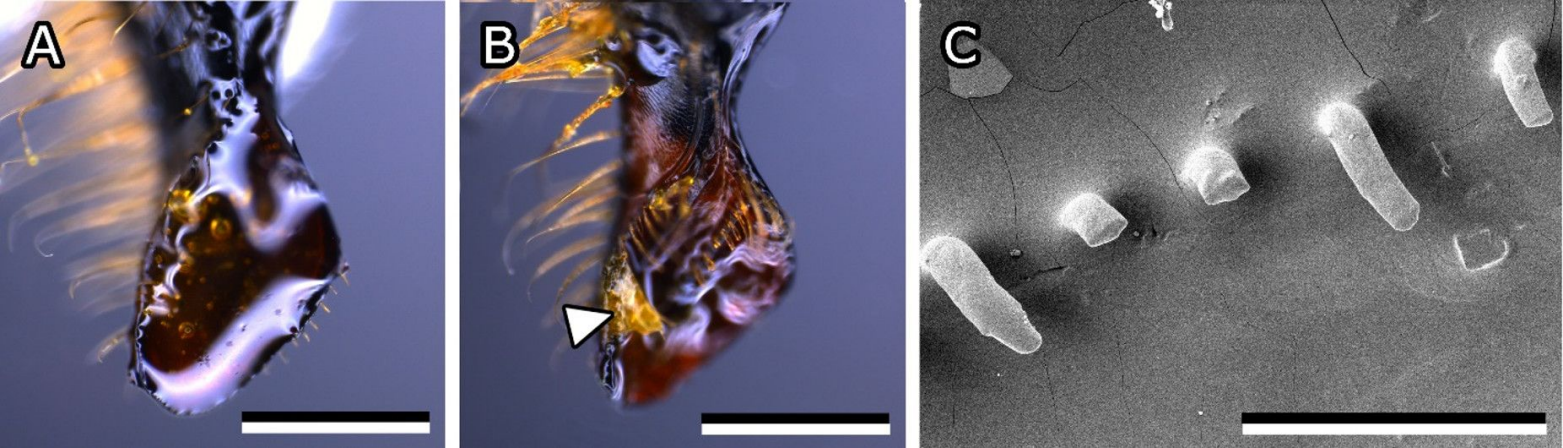
Mandibles of propolis bees. (A) Mandible tip completely covered with resin. (B) Resin residues on mandible. The arrowhead points to resin rests adhering to the hairs on the mandible. (C) SEM micrograph of central ridge and bristles covered by resin. Scale bars: 500 µm (A,B), 100 µm (C).

For the sake of distinction, we refer to bees that were caught when they returned to the hive with resin attached to their corbiculae as “propolis bees”. Their untreated mandibles were visually examined to compare them to regular worker bees in terms of morphology and contaminations. Mandibles of propolis collecting bees exhibited the same form and features as those of regular worker bees, though they were covered with resin to varying degrees. Some mandibles were completely filled with resin (Figure 6A), while others only had resin remnants stuck in hairs (Figure 6B). However, all types of resin contaminations were easily removed with fine forceps and no resin residues were left behind. Bristles along the central ridge of propolis bees were often more damaged compared to regular worker bees (Figure 4A and Figure 6C). Especially the tips were regularly blunt or broken off. Untreated mandibles of regular worker bees that were not observed collecting propolis were often contaminated with small crumbs and pollen particles but not with resin.

### Propolis adhesion on bee mandibles

The adhesion of propolis samples on several untreated bee mandibles (BM) was measured to determine whether mandibles of bees possess anti-adhesive properties. No statistical differences were found between work of adhesion values of single measurements that were conducted on different positions of the bee mandible (*P* = 0.995). For some measurements, near the hairy edge of the bee mandible the point of contact was not clearly distinguishable in the loading curve due to hair touching the sample before it contacted the cuticle. However, in the unloading curve the adhesive failure (pull-off) was easy to identify in most cases.

**Figure 7:**
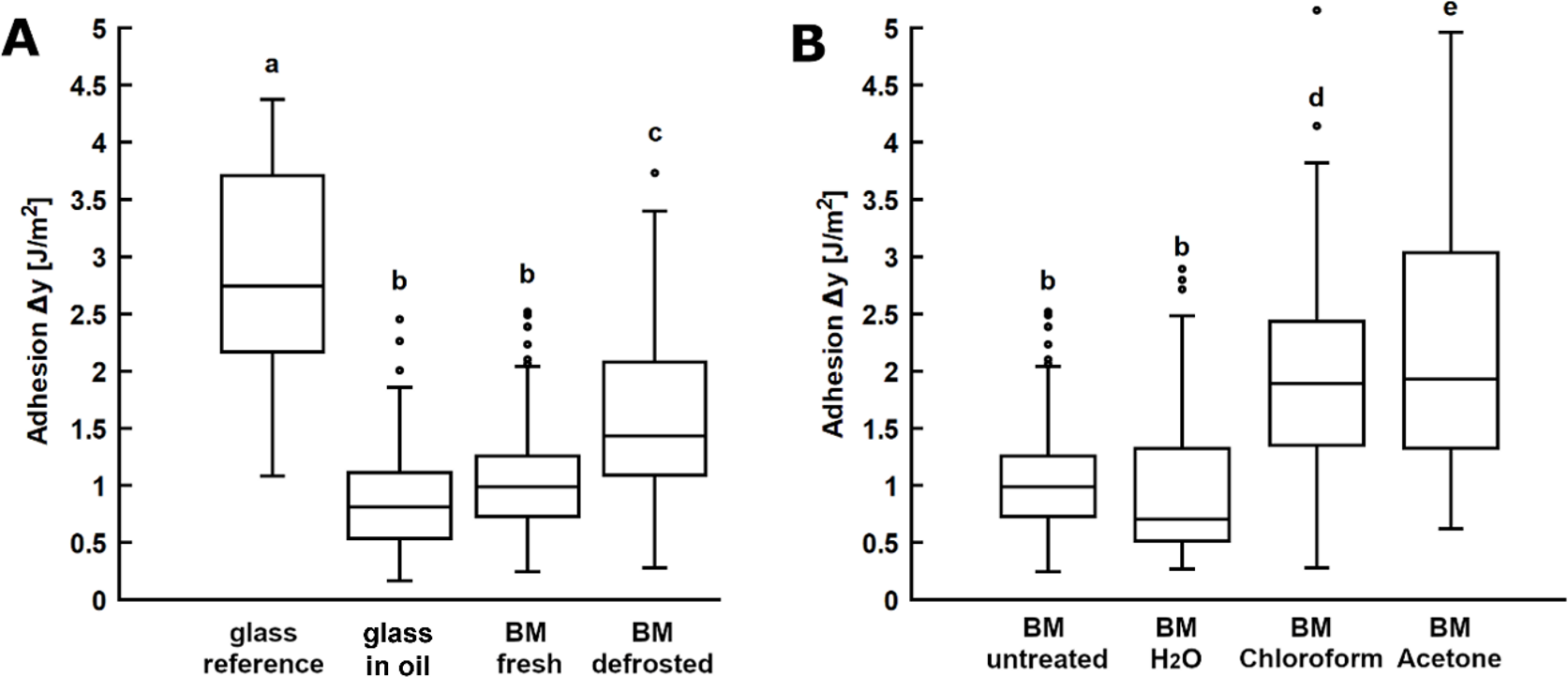
Propolis adhesion on bee mandibles. (A) Adhesion of propolis on bee mandibles compared to glass. Adhesion experiments with propolis samples were carried out on the following substrates: a dry glass surface (glass reference), a glass surface with a drop of oil (glass in oil), freshly prepared, untreated mandibles (BM fresh), defrosted and subsequently prepared mandibles (BM defrosted). (B) Adhesion on bee mandibles washed with different methods. Adhesion experiments with propolis samples were carried out on the following substrates: freshly prepared, untreated mandibles (BM untreated), freshly prepared mandibles washed with water (BM H_2_O), prepared mandibles washed with chloroform and water (BM chloroform), prepared mandibles washed with acetone and water (BM acetone). All experiments were conducted using the micro-force tester Basalt-01 at room temperature (24 °C) with a maximum applied force of 5 mN (*N* = 5–8 propolis samples per condition, *n* = 10 individual measurements per sample). The ends of the boxes define the 25th and 75th percentiles, with a line at the median and error bars defining the 10th and 90th percentiles. Conditions marked with different letters differ significantly from each other (one-way ANOVA, *P* < 0.001 and Tukey test, *P* < 0.05).

In contrast to the pull-off force, the work of adhesion does not depend on the radius of the sample. Therefore, the work of adhesion value is hereafter used to compare adhesion from different adhesion experiments. Adhesion of propolis on fresh and previously frozen bee mandibles was compared to the data obtained on glass in dry and fluid conditions (in oil) (Figure 7A, Table 1). An analysis of variance (ANOVA) was performed to find out, whether statistical differences exist between adhesion values on these surfaces. The *P*-value was found to be smaller than the significance level α = 0*.*001. Therefore, a posthoc Tukey test was conducted to find pairwise differences. *P*-values smaller than α = 0*.*05 were found to be significant.

**Table 1:** Propolis adhesion on mandibles. Work of adhesion and pull-off forces obtained from adhesion experiments conducted using Basalt-01 mechanical tester (Tetra GmbH, Ilmenau, Germany). If not stated differently, tests were carried out at room temperature (24 °C) with a set normal force of 5 mN and a contact time of 0 s or 60 s (*N* = 5–15 propolis samples and corresponding mandibles per condition, *n* = 10 individual measurements per sample). Mean values and standard deviations (s.d.) are given. Reference measurements on glass in dry and wet conditions were reported by [1]. BM, bee mandible.

		Work of adhesion [J*/*m^2^]		Pull-off force [mN]	
Substrate	N*n	mean	s.d.	mean	s.d.

reference (dry glass)	80	2.96	1.27	2.12	0.77
reference (glass in oil)	60	0.90	0.55	0.74	0.36
BM, fresh	150	1.01	0.21	0.71	0.33
BM, defrosted	50	1.66	0.51	1.23	0.45
BM, water-washed	50	0.99	0.53	0.91	0.51
BM, chloroform-washed	100	1.87	0.49	1.45	0.77
BM, acetone-washed	50	2.14	1.20	1.58	0.55
BM, fresh, 60 s	5	2.66	0.42	2.70	0.81
BM, chloroform-washed, 60 s	10	4.20	1.12	3.37	0.91
BM, acetone-washed, 60 s	5	3.38	1.19	2.58	0.32
BM, replica	60	3.29	1.19	2.84	0.62

The mean work of adhesion on fresh, untreated mandibles was measured to be 1.01 ± 0.21 J*/*m^2^ (pull-off force: 0.71 ± 0.33 mN), which is significantly lower than adhesion on dry glass (2.96 ± 1.27 J*/*m^2^, *P* < 0*.*0001) (as well as all other analysed dry technical surfaces) [1]. Moreover, work of adhesion on fresh mandibles of bees was comparable to adhesion measured on glass in the presence of fluid (mineral oil) (0.90 ± 0.55 J*/*m^2^, *P* = 1.0) [1].

There were no significant differences between adhesion on fresh mandibles caught and tested in October and July (*P* = 0.9999). Compared to fresh mandibles, propolis adhered significantly stronger to mandibles that had been stored in the freezer (−20 °C) for three months prior to the experiment (1.66 ± 0.51 J*/*m^2^, *P* = 0.0613).

Compared to the work of adhesion on fresh bee mandibles where the propolis sample was retracted as soon as the maximum load was reached, the work of adhesion increased significantly to 3.45 ± 1.44 J*/*m^2^ (*P* < 0*.*0001) when the contact time of the propolis sample was increased to 60 s.

#### Surface coating on bee mandibles

**Figure 8:**
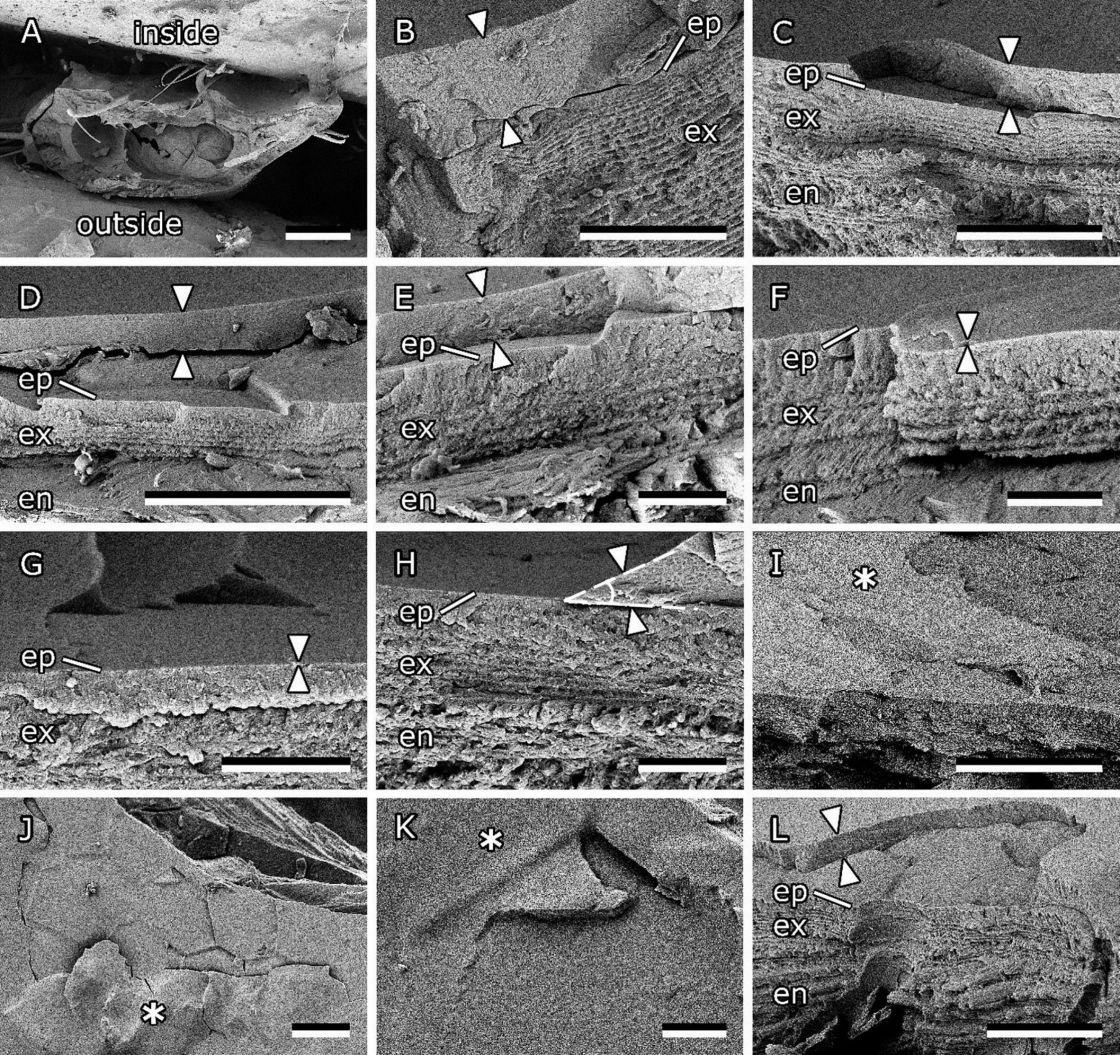
Fractures of freshly prepared, frozen bee mandibles. (A) Overview of mandible fracture. Inside and outside are marked. (B) Cuticle in channel area. (C–F) Cuticle in the flat area. (G,H) Cuticle near the sharp edge. (H) Contact angle of surface coating on cuticle (25°). (I–K) Angled top view of flat area. (L) Cuticle on outside of mandible. ep, epicuticle; ex, exocuticle; en, endocuticle. Arrowheads indicate the surface layer on top of the cuticle. Asterisks mark the top view of the surface layer. Scale bars: 100 µm (A), 10 µm (B–D,I,J,L), 2 µm (E–H,K).

**Figure 9:**
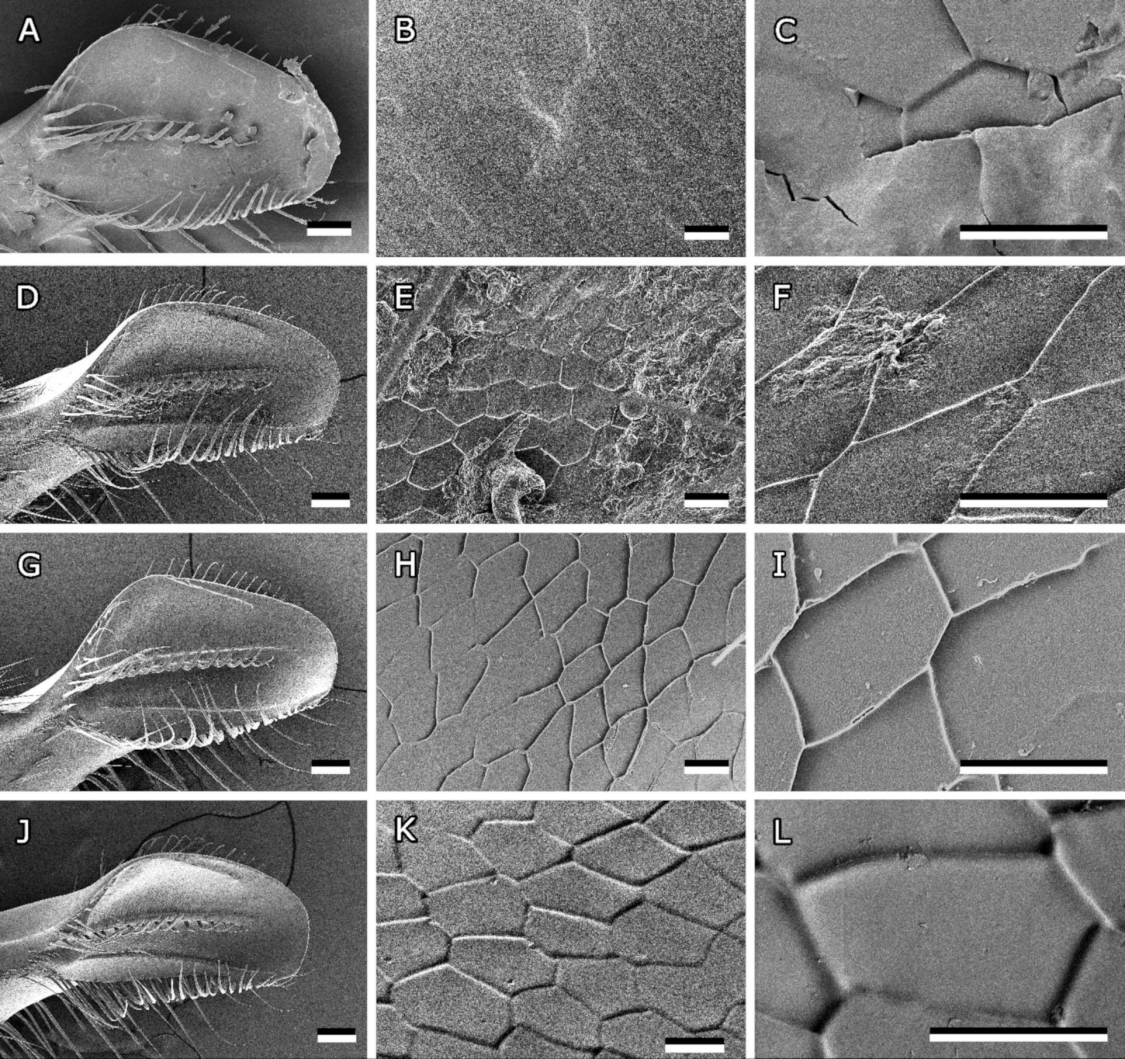
Bee mandibles washed with different methods. (A–C) Cryo-SEM micrographs of untreated bee mandibles (overview and close-up of flat area). (D–F) SEM micrographs of bee mandibles washed with water. (E) channel area, (F) flat area. (G–I) SEM micrographs of bee mandibles washed with acetone and water (mandible overview and close-up of flat area). (J–L) SEM micrographs of bee mandibles washed with chloroform and water (mandible overview and close-up of flat area). Scale bars: 100 µm (A,D,G,J), 10 µm (B,C,E,F,H,I,K,L).

To figure out possible reasons for low propolis adhesion on bee mandibles, freeze fractures of freshly prepared mandibles were studied in the cryo-SEM (Figure 8). The different layers of the cuticle (endo-, exo-, and epicuticle) were clearly distinguishable on fractured samples. On fresh mandibles, an additional, possibly fluid, layer was present on top of the epicuticle. The layer thickness varied depending on the area of the mandible. The layer was thickest (8.37 ± 3.32 µm) in the channel between the ledge and central ridge (Figure 8B). In the flat area the layer measured 1.56 ± 1.04 µm in thickness (Figure 8C–F). Close to the unstructured sharp edge the layer was thin (0.053 ± 0.015 µm) (Figure 8G,H). In some places the contact angle of the surface layer on the cuticle could be measured on SEM images (Figure 8H). Varying angles between 8° and 30° were found. On the medial surface of the frozen mandible the substance overspreading the surface partially hid the scales, especially in the channel area (Figure 9A–C). Residues of this substance were still visible in dried specimens studied in regular SEM.

**Figure 10:**
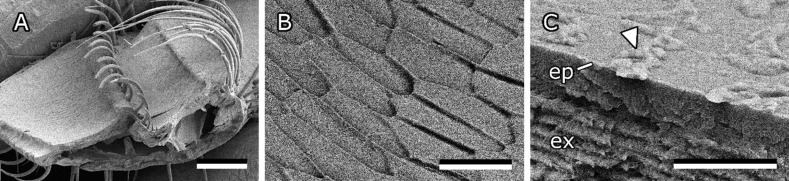
Fractures of bee mandibles washed with chloroform. (A) Cryo-SEM micrograph of fractured bee mandible. (B) Cryo-SEM micrograph of scales on washed mandible. (C) Cryo-SEM micrograph of fractured cuticle. Arrowheads indicate residues on epicuticle. ep, epicuticle; ex, exocuticle. Scale bars: 100 µm (A), 10 µm (B), 2 µm (C).

Different washing treatments were applied in order to remove the surface layer from the mandibles. Examination in the SEM showed that on mandibles washed with water the residues of the substance covering the surface were still present to a similar extent as on untreated mandibles studied with the same method (Figure 9D–F). On mandibles washed with acetone (Figure 9G–I) and chloroform (Figure 9J–L) the majority of the surface coating was removed. Though, cryo-SEM studies of chloroform-washed mandibles showed that minor remnants of the surface coating remained on some areas of the epicuticle even after washing (Figure 10).

#### Adhesion on washed bee mandibles

Adhesion of propolis was tested on washed bee mandibles and compared to adhesion on untreated specimens (Figure 7B, Table 1). Treatment with water alone had no significant effect on propolis adhesion on mandibles. The work of adhesion on mandibles washed with water was measured to be 0.99 ± 0.53 J*/*m^2^ (*P* = 1.0). Compared to fresh, untreated mandibles, a significantly higher work of adhesion of 1.87 ± 0.49 J*/*m^2^ (*P* = 0.0014) was measured on mandibles washed with chloroform and water and an even higher work of adhesion of 2.14 ± 1.20 J*/*m^2^ (*P* < 0*.*0001) on mandibles washed with acetone and water. The work of adhesion of propolis on washed bee mandibles was significantly higher, when the contact time was increased to 60 s and compared to values for 0 s (chloroform-washed: *P* < 0*.*0001, acetone-washed *P* = 0.0236). Comparing the work of adhesion obtained from tests with a 60 s contact time performed on untreated and washed mandibles shows a significantly higher propolis adhesion on washed mandibles (*P* < 0.0001).

#### Artificial bee mandible

**Figure 11:**
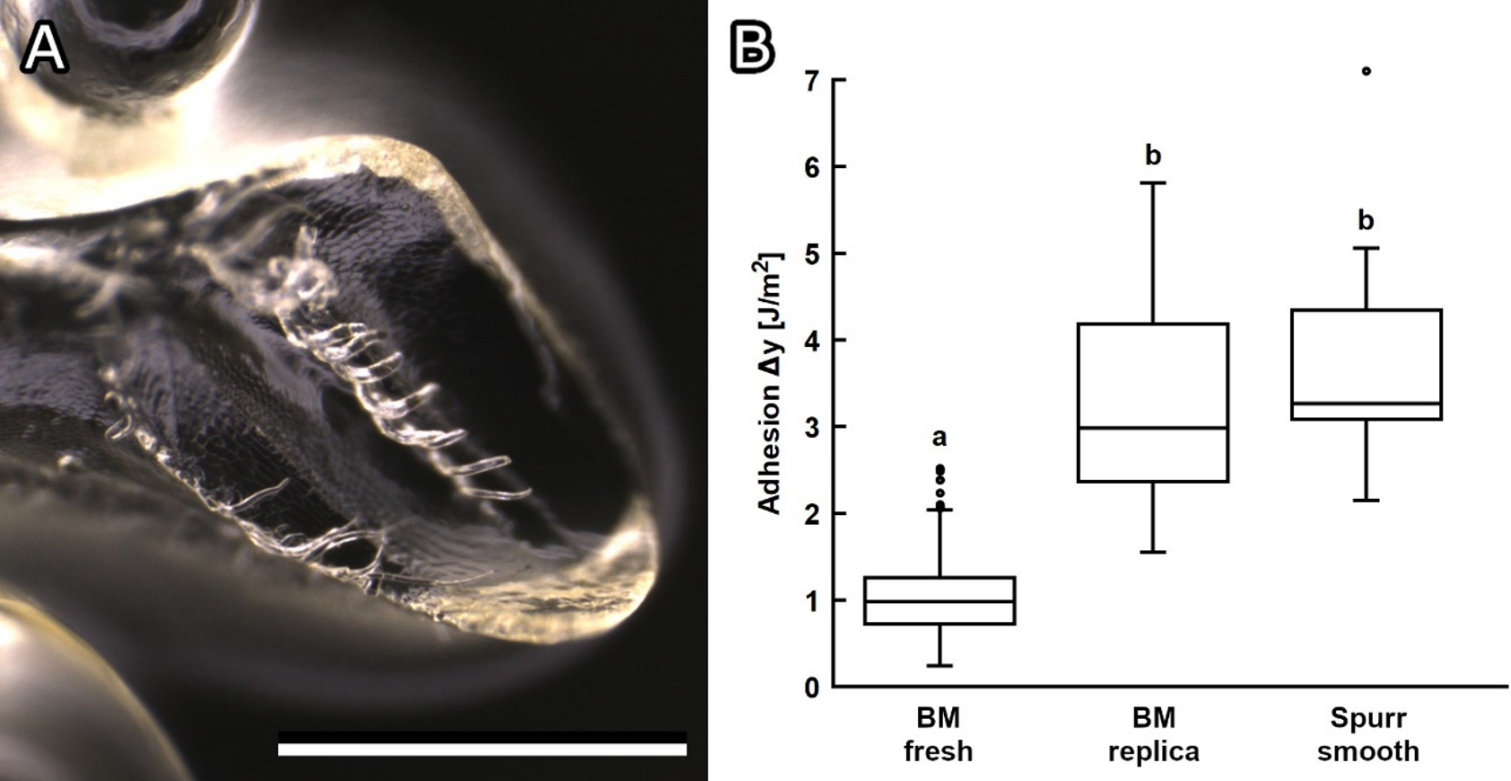
Replica of bee mandible. (A) Bee mandible replica made of Spurr’s epoxy resin. (B) Propolis adhesion on real and replicated bee mandibles and smooth resin surface. The adhesion experiments were conducted using the micro-force tester Basalt-01 at room temperature (24 °C) with a maximum applied force of 5 mN (*N* = 5–8 propolis samples per condition, *n* = 10 individual measurements per sample). The ends of the boxes define the 25th and 75th percentiles, with a line at the median and error bars defining the 10th and 90th percentiles. Conditions marked with different letters differ significantly from each other (one-way ANOVA, *P* < 0.001 and Tukey test, *P* < 0.05). Scale bar: 500 µm (A).

In order to test the effect of the surface structures and topography of the mandible as well as material properties on propolis adhesion, further adhesion experiments were performed. The morphology of real mandibles including the microstructures were successfully replicated in mandible replica made from Spurr’s epoxy resin (Figure 11A). Propolis adhesion was subsequently tested on these replicated mandibles and compared to adhesion on the smooth Spurr substrate and fresh bee mandibles (Figure 11B, Table 1). There was no significant difference between propolis work of adhesion on replicated resin mandibles (3.29 ± 1.19 J*/*m^2^) and on a smooth surface of Spurr’s resin (*P* = 1.0). Adhesion on fresh bee mandibles was however significantly lower than on both substrates made from Spurr’s resin (*P* < 0.0001).

## Discussion

### Anatomy of honeybee mandibles

A honeybee mandible is a highly specialised organ that is used for multiple purposes such as feeding, defence, and propolis foraging and processing [6–7]. Using their mandibles, honeybees can handle propolis, apparently without being negatively affected by the sticky material. The anatomy of the mandible was therefore studied to understand its interaction with propolis. The stable construction of the mandible, especially the thick stem, is well suited to handle tough material like propolis. The medial surface of the mandible is in particularly close contact with propolis during processing and was therefore examined more closely. The medial surface of the spoon-shaped mandible tip was previously described as ridged and concave [8]. We found that there were several ridges and ledges on the medial surface of the mandible forming a channel between them. A hairless and sharp edge on one side could be used to cut materials as it has been suggested for the mandible of stingless bees [19]. Long flexible hairs grow on one edge and reach over half of the mandible. Stiff bristles spike the centre ridge. These hairs could play a role in cleaning as they could help to brush off contamination.

#### Surface structures on bee mandibles

Microstructures were found on the medial surface of honeybee mandibles. These anisotropic structures looked like scales and their proportions and orientation differed depending on the location on the mandible. The structures faded and eventually disappeared towards the apex and hairless edge of the mandible. It is possible that the scales in these areas are worn out by intensive use. However, it can be assumed that this is the normal morphology of the mandibles, as all examined mandibles had this characteristic. Anisotropic structures are also present on other animals such as snakes and were proposed to support anisotropic properties, for example, anisotropic friction [34]. The scales on bee mandibles are mostly oriented towards the apex and might help the bee to remove contaminations and maybe even help to handle and form sticky substances. Similar scale patterns are found on many insects [35], for example, in the trochanter of ants [19]. In contrast to honeybee mandibles, no microstructures were found on the medial surface of stingless bee mandibles [19].

#### Mandibles of propolis bees

Mandibles of propolis bees exhibited the same features as mandibles of regular worker bees. They were however frequently contaminated with varying amounts of resin. This shows that bees do not seem to be able to completely prevent resin adhesion. Nevertheless, resin residues were easy to remove from the mandibles and did not adhere strongly to the mandible surface. Worker bees that were not observed foraging resin did not show similar contaminations. This could indicate that bees are able to clean their mandibles and get rid of resin contaminations. But bees exhibit a division of labour and older bees are believed to be responsible for resin foraging [10]. Therefore, it cannot be assumed that all bees collected for adhesion tests have already been in contact with resin during their lifetime. An observation of propolis bees in the hive would be helpful to find out more about cleaning behaviour and effectiveness. This could prove to be difficult as propolis bees are rarely observed even outside the hive. The bristles on mandibles of propolis bees were often more damaged than those of normal workers. This could be a simple sign of aging or a consequence of increased stress during the processing of resin.

### Propolis adhesion on bee mandibles

It has been reported [2] that propolis is a very sticky substance. Previous studies showed that propolis work of adhesion on glass is approximately 3 J/m^2^ (Table 2, [1]). We performed adhesion experiments with propolis on the medial surface of bee mandibles to understand how bees are able to interact with this substance. Low propolis adhesion (1 J/m^2^) occurred on fresh bee mandibles compared to technical surfaces such as glass, steel, PTFE, or Spurr resin (Table 2, [1]). Propolis work of adhesion on bee mandibles was of a similar magnitude to that measured on glass under fluid conditions (0.9 J/m^2^ [1]). It was shown before that the adhesion of propolis does not show a great dependence on the substrate material [1]. It is therefore unlikely that the surface chemistry of the bee cuticle alone is responsible for the low adhesion. Possible mechanisms to reduce adhesion could be surface microstructures, an easy-to-break solid layer preventing strong bonding, or a fluid layer providing cohesion failure [16]. The adhesion on mandibles stored in the freezer for a longer period was higher than on those freshly prepared. This could indicate that an at least partially volatile substance on the mandible reduces adhesion of propolis. Similar adhesion was measured in all areas of the bee mandible. However, the exact contact area was hard to control and see during testing, making it difficult to compare positions.

**Table 2:** Propolis adhesion on different substrates. Table 2 was reproduced from [1] (© 2021 Saccardi, Schiebl, Weber, Schwarz, Gorb and Kovalev, published by Frontiers, distributed under the terms of the Creative Commons Attribution 4.0 International License, https://creativecommons.org/licenses/by/4.0).

Substrate	*N*·*n*	Work of adhesion [J/m^2^]			Pull-off force [mN]	
		
		mean	s.d.		mean	s.d.

Glass	80	2.96	1.28		2.12	0.77
PTFE	50	2.91	0.71		1.43	0.72
Steel	50	2.29	0.82		1.98	0.51
Spurr	50	3.61	0.95		3.35	0.87

#### Surface coating on bee mandibles

Freeze fractures of bee mandibles clearly showed the construction of the bee mandible. Like the cuticles of most insects, it consists of endocuticle, exocuticle, and epicuticle [36]. Fractures also revealed that an additional, possibly fluid, layer is present on top of the mandibular epicuticle. From the cryo-SEM images similar to that in Figure 8H it is even possible to determine the contact angle of the fluid on the epicuticle. This substance has a relatively small contact angle (<30°) on the cuticle and is therefore likely to easily spread across the mandible surface. The layer was found to be thicker in the channel area and thinner towards the apex and hairless edge of the mandible.

#### Effect of surface coating on propolis adhesion

Our results suggest that above-mentioned layer reduces propolis adhesion, as adhesion is very low on fresh, untreated mandibles but significantly higher on mandibles washed with acetone or chloroform. SEM analysis also showed that the fluid layer was mostly removed after treatment with either acetone or chloroform. The fluid probably prevents direct contact between propolis and the mandible surface as described previously for other insects [16]. When the bee processes propolis, it only comes into contact with the liquid on the mandible surface and as the contact is removed, cohesive failure occurs. Part of the fluid sticks to the propolis sample, while the rest remains on the epicuticle. When longer contact occurs between mandible and propolis, the material has time to relax and adapt to the surface and probably to displace the fluid layer in some areas, leading to higher adhesion. The fact that the surface coating on the mandible can be removed with acetone or chloroform but not water suggests it might be a waxy or oily substance [16,37]. The low contact angle (<30°) of the substance suggests that the mandible surface might be oleophilic. This is consistent with observations that insect cuticles are generally rather wetted by oils than by water [38]. There are different possibilities for the origin of the lubricating layer. It has previously been suggested that the fluid secreted by the mandibular gland might help to reduce adhesion of propolis and waxes on mandibles [7,39]. For stingless bees, lubricating substances were suggested to originate from mandibular, salivary, or cephalic glands [19,40]. It was also suggested that due to gland development, only older stingless bees are able to handle propolis [20].

Mandibular glands of honeybees have been found to have a high potential to produce lipids [41]. Glucose oxidase, produced in the mandibular glands, has been found in propolis [42–43]. Which could be an indicator of bees handling propolis with mandibles covered in mandibular secretion. The opening of the mandibular gland was not visible on our micrographs, but it has been described to open on the inside of the mandible base (proximal end) [7]. The fluid might be guided from the mandibular gland to the mandible tip through the groove on the stem of the mandible and then through the channel on the medial surface of the mandible tip. This groove was previously suggested to channel liquid to the mandible tip [6,19]. Bees possibly help the spreading of fluid across the mandible surface by moving and rubbing their mandibles against each other as they do during grooming [44]. In [19], it was also suggested that regurgitated nectar or honey could be used to lubricate mandibles of stingless bees. Another possible origin of the adhesion-reducing fluid on mandibles might be secretion through pore canals as the cuticle of insects often contains pore canals that transport lipid-containing secretions [45]. Cuticular lipids such as alkanes, alkenes, and fatty acids are present on the head and other body parts of honeybees [46]. Their functions include nestmate recognition and protection against desiccation [46]. More alkanes have been found on the bodies of forager bees compared to nursing bees [47].

#### Effect of the mandible surface structure on adhesion

In some cases, it has been described that microstructures can reduce adhesion by decreasing the contact area [48–49]. The trochanter of ants (*Camponotus sericeiventris*) is structured with scales that were suggested to lower adhesion [19]. Resin adhesion on these scales was significantly lower than on the unstructured mandibles of stingless bees [19]. As scale-like microstructures were found on honeybee mandibles, their effect on propolis adhesion was tested. For this purpose, the form of the mandibles, including the scale-like microstructures, was replicated in resin in order to be able to have a direct comparison between propolis adhesion on the same material in mandible and smooth form. Propolis adhesion on the mandible replica did not differ significantly from that on a smooth resin control surface. It, therefore, seems like the structures on mandibles do not directly affect propolis adhesion. It is, however, likely that the scales help to spread the fluid over the surface to create an even layer. Structures also prevent the fluid from flowing off easily. In unstructured areas of the mandible, the layer was thinner. It has previously been described that structures facilitate the spreading of fluid [50]. For instance, structures on *Nepenthes alata* and lizard skin have been found to enhance unidirectional liquid transportation [51]. The contact area of propolis in our adhesion experiments was smaller than in reality when a bee handles propolis. On a bigger scale, hair and overall topography of the mandible could also reduce propolis adhesion, as they might prevent full contact with the mandible surface.

#### Outlook

Reduced propolis adhesion was observed on the surface of bee mandibles. The anti-adhesive strategy found in bees might also be helpful in solving technological adhesive problems, for instance, unwanted resin contamination of woodworking tools.

The next step in the further course of the top-down development process is the attempt to further understand the principle of the presumed anti-adhesive strategy of the honeybee and to verify it in replicas. The abstraction of the principle or structures found and the transfer to the technosphere with an effectiveness test under technical production processes are the next steps in the top-down procedure.

## Conclusion

Studying the interaction between propolis and bee mandibles revealed that propolis adhesion is indeed reduced on bee mandibles. Propolis adhesion is four times reduced in the presence of a natural secretion layer on top of the mandibular epicuticle, while the scale-like micropattern on the mandible might help the spreading of the fluid. The lubricating layer could be removed with organic solvents. In addition, the hair on mandibles is likely to help in the cleaning process.
